# Patients readmitted to the intensive care unit: can they be prevented?

**DOI:** 10.1186/1755-7682-6-18

**Published:** 2013-04-27

**Authors:** Shahla Siddiqui

**Affiliations:** 1 Khoo teck Puat Hospital, Singapore, Singapore

## Abstract

Readmission to the surgical intensive care unit of a tertiary care hospital has traditionally been tracked as a quality indicator and many studies have suggested various figures as to the acceptable rate of such. ICU beds being a precious resource readmitting a patient could imply hasty discharge or inadequate care. Patients readmitted generally have a higher mortality and length of stay due to the worsening of their illness. The definition of ‘ICU readmission’ varies from either in the first 24 hours, to over the next 2 days or even whether the patient comes back during the entire period of admission. The association between increasing severity of illness and the risk of readmission to ICU has not been systematically summarized and one can speculate as to the various predictive signs of possible readmission. We looked at our data over the past 5 months of all adult surgical ICU patients who were readmitted during the same admission after ICU discharge. Fourteen patients were readmitted with the monthly rate varying from 3-11% per month. The age ranged from 33 to 90 years and the gender was mostly male. The patients’ initial admission diagnosis varied as they belonged to General surgery, ENT, Neurosurgery and Orthopedic disciplines and the time from initial discharge to readmission ranged from 40 to 4 days. The majority of the readmission causes were respiratory and these included desaturation, PE, pneumonia and mucus plugging. Other causes included hypotension, sepsis, dysrhythmias, recurrent drop in GCS and GI re-bleed. When compared to the first admission most patients had a longer length of stay during the readmission. The outcomes were mostly good with only one patient expiring after readmission.

## 

Dear Sir,

Readmission to the surgical intensive care unit of a tertiary care hospital has traditionally been tracked as a quality indicator and many studies have suggested various figures as to the acceptable rate of such [1]. ICU beds being a precious resource readmitting a patient could imply hasty discharge or inadequate care. Patients readmitted generally have a higher mortality and length of stay probably due to a graver prognosis [2]. The definition of ‘ICU readmission’ varies from either in the first 24 hours, to over the next 2 days or even whether the patient comes back during the entire period of admission. The association between increasing severity of illness and the risk of readmission to ICU has not been systematically summarized in the surgical ICU population and one can speculate as to the various predictive signs of possible readmission [3,4]. Frost [5,6] and Rosenberg et. al [7] in their work have looked at some of these factors however not focused in the surgical ICU. Frost et. al have presented methods to develop hospital specific risk models but not SICU focused, to identify patients at high risk of readmission to ICU [6].

We looked at our data over the past 5 months of all adult surgical ICU patients who were readmitted during the same admission after ICU discharge. Fourteen patients were readmitted with the monthly rate varying from 3-11% per month. The age ranged from 33 to 90 years and the gender was mostly male. The patients’ initial admission diagnosis varied as they belonged to General surgery, ENT, Neurosurgery and Orthopedic disciplines and the time from initial discharge to readmission ranged from 40 to 4 days. The majority of the readmission causes were respiratory and these included desaturation, PE, pneumonia and mucus plugging. Other causes included hypotension, sepsis, dysrhythmias, recurrent drop in GCS and GI re-bleed. When compared to the first admission most patients had a longer length of stay during the readmission. The outcomes were mostly good with only one patient expiring after readmission to the ICU.

In trying to learn lessons from this data one can say that optimizing organ functions in these patients before discharge from the ICU could result in reduced readmission rates, however our results show that the patients are a very heterogeneous group and apart from respiratory compromise there is no single factor that may predict their chances of being readmitted. Delaying discharge in ICU patients can be a waste of resources and deny the chance to other patients of an ICU bed who may be in dire need of critical care [7]. We therefore suggest that perhaps patients can be ‘flagged’ as needing more respiratory or physical therapy, closer nursing in step down units, continuation of respiratory care bundles in the ward, use of incentive spirometry and nebulisation routinely in the wards as well as head of bed elevation and aspiration precautions to reduce the rate of aspiration pneumonia. DVT prophylaxis as well as GI prophylaxis may attenuate the chances of PE and GI bleed in such patients who may need a closer watch than routine ward patients. With these endeavors we may prevent unwanted ICU readmissions ….or will we? That remains to be seen.

Thanking you

Regards

Dr. Shahla Siddiqui

Khoo teck Puat Hospital,

Singapore.

## Competing interests

The authors declare that she has no competing interests.
